# Maxillofacial metastasis from breast cancer

**DOI:** 10.11604/pamj.2014.19.156.4948

**Published:** 2014-10-15

**Authors:** Tariq Namad, Zineb Benbrahim, Rajae Najib, Afif Mohammed, Soufiane Baggar, Nezar Bouyahia, Samia Arifi, Nawfel Mellas

**Affiliations:** 1 Department of Medical Oncology, Hassan II University Hospital, Fez, Morocco; 2 Department of Radiation Therapy, National Institut of Oncology, Rabat, Morocco

**Keywords:** Breast cancer, maxillofacial, metastasis

## Abstract

Metastatic tumors to paranasal sinuses are exclusively rare. In this paper, we report acase of breast carcinoma metastasizing to the right maxilla. The metastasis occurred 5 years after radical mastectomy and presented as a primary sinonasalmass. The diagnosis was confirmed with histopathologic andimmunohistochemical examination however the patient died before starting any specific treatment because of tumor bleeding.

## Introduction

Metastatic tumors in the maxillary sinus are uncommon. The first cases reported in the literature were described by Bernstein et al. in 1966. Since then few cases have been published. In this paper, we are describing a case of metastatic breast cancer in the right maxillary sinus and we review different data about diagnosis and treatment of this entity.

## Patient and observation

**Figure 1:**
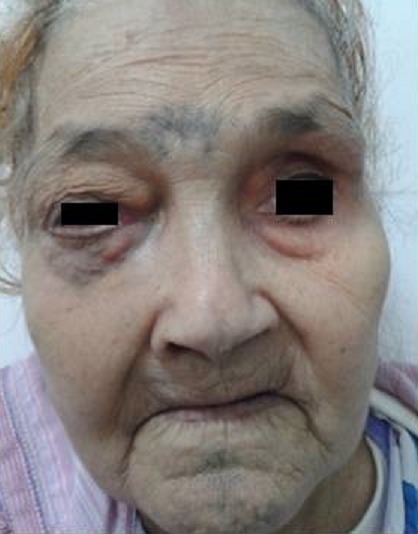
Clinical presentation of the maxillary metastasis from breast cancer (right facial mass, ptosis and collateral veinous circulation)

**Figure 2:**
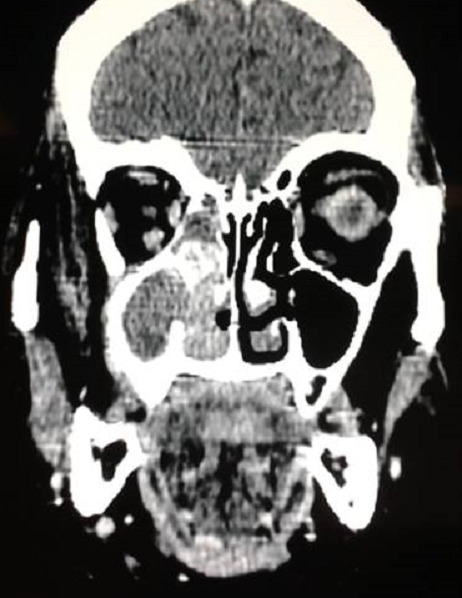
Radiological presentation of the maxillary metastasis from breast cancer (solid mass occupying the right maxillary with orbital and endonasal extension)

In June 2007, a 72-year-old female with history of diabetes was diagnosed as having a grade 2, hormone receptors positive, HER2 negativeinfiltrating ductal cancer of the left breast. Left radical mastectomy with axillary regional lymph node dissection was performed. Three lymph nodes were involved among twelve analyzed. The patient underwent adjuvant chemotherapy, radiotherapy and tamoxifen based endocrinotherapy for 5 years. In February 2013, thepatient experienced a right-sided headache without nasal obstruction andepistaxis. One month later, a right facial mass, causing decreased visual acuity, ptosis and collateral venous circulation in the periorbital region were clinically noted (Figure 1). The Rhinoscopy revealed normal respiratory mucosa. The craniofacial CT objectified a solid mass occupying the right maxillary with orbital and endonasal extension (Figure 2). The sinonasal biopsies confirmed the diagnosis of metastasisfrom breast cancer with positivity of hormone receptors. The complementary work up revealed pulmonary metastases. No other distant metastases were revealed on abdomen and pelvis CT scan. The patient was referred to the Department of medical oncology of Hassan II University Hospital for palliative chemotherapy, however the patient died of tumor hemorrhage before starting specific treatment.

## Discussion

Metastatic tumors involving the head and neck region are very uncommon, nevertheless, metastatic diseases occurring in the nose and paranasal sinuses are exclusively rare [1–3] and not exceeding 1% of all malignant tumors produce metastatic foci in the jaws [4]. The most known tumors to have a potential to give metastases in this area are renal cancer [5], followed by testicular tumors, bronchial cancer, gastrointestinal neoplasm and breast cancer [3]. The metastatic breast cancer can also give metastases in other sites of the facial maxillary area such as the sphenoidal sinus [6], frontal sinus [7, 8] and ethmoidal [9, 10], even more in the vascular channels [9]. Thedistant spread is unusual before the disease spreads locally, and distant metastases are uncommon in the absence of lymph node metastases [11]. Whilst, some authors [12] showed the role of the vertebral venous plexus in tumor spread. Other authors showed that ethmoidal involvement by metastatic carcinoma might be due to direct transcribrosal [3, 13]. The metastatic tumors to the paranasal sinuses have no distinctive clinical features,it may be asymptomatic or may presented as facial pain, epistaxis, nasal obstruction and facial asymmetry as the main manifestations [5, 13] and they can mimic a primary facial cancer. Nevertheless, diplopia, epiphora, blepharoptosis, decreased visual acuity and proptosis are the main clinical symptoms when the metastatic tumor develops in the orbit [14–16]. Surgical excision isstill the preferred treatment, unfortunatelythis therapeutic approach cannot be radical, surgery usually being limited to obtaining a tumor biopsy for differential diagnosis [3] and curative methods to treat such tumors have not met much success [16].

The objective of treatment of these patients is to improve or maintain their quality of life. However, theonly palliation is possible since there is either local spread or distant metastasis to other organs [11]. Preventing probable bleeding and pain relief should also be a part of the main goal of treatment [16]. Local radiotherapy is almost always the treatment of choice for pain relief, preventing tumor growth and improving function of the organ [11, 17, 18]. The prognosis for patients with metastasis to paranasal sinuses is generally poor [18]. In fact, a review of the literature showed that all those patients presenting this finding died some months after diagnosis [3]. In our case the patient died after 2 months after metastasis diagnosis.

## Conclusion

In summary, we experienced a case of breast cancer metastasizing in right maxillary sinus 5 years after initial treatment. This case showed a poor prognosis of this localization with death of the patient because of tumor hemorrhage.
